# Systematic review exploring human, AI, and hybrid health coaching in digital health interventions: trends, engagement, and lifestyle outcomes

**DOI:** 10.3389/fdgth.2025.1536416

**Published:** 2025-04-24

**Authors:** Croía Loughnane, Justin Laiti, Róisín O’Donovan, Pádraic J. Dunne

**Affiliations:** Centre for Positive Health Sciences, Royal College of Surgeons in Ireland, Dublin, Ireland

**Keywords:** digital health interventions, positive health, health promotion, disease prevention, coaching, health coaching

## Abstract

**Introduction:**

Digital Health Interventions (DHIs) have been identified as a solution to the United Nations Sustainable Development Goals (SDG3) for health promotion and prevention. However, DHIs face criticism for shallow and transactional engagement and retention challenges. Integrating DHIs with health coaching represents a promising solution that might address these issues by combining the scalable and accessible nature of DHIs with the meaningful and engaging nature of health coaching. This systematic review aims to synthesise existing peer-reviewed research on coach-facilitated DHIs to understand how digital health coaching is being used in DHIs and the impact it has on engagement and lifestyle outcomes.

**Methods:**

Studies examining DHIs with a coaching component addressing lifestyle outcomes were included. A search of APA PsychINFO, Medline, Web of Science, and Scopus was performed from inception to February 2025. Three authors conducted the study selection, quality appraisal using the Mixed Methods Appraisal Tool (MMAT), and data extraction. Data extraction captured study characteristics, coaching features, participant engagement, and lifestyle outcomes.

**Results:**

Thirty-five studies were identified and synthesised using a narrative synthesis approach. This review highlights three coaching modalities in DHIs: digital human coaching, Artificial Intelligence (AI) coaching, and hybrid (human-AI) coaching. All coaching modalities demonstrated feasibility and acceptability.

**Discussion:**

While both human and AI coaching have shown a positive impact on both engagement and lifestyle outcomes, hybrid approaches need further refinement to harness AI's scalability and the depth of human coaching. However, the variability of engagement metrics and coaching protocols limited study comparability. Standardising how engagement and coaching delivery are measured and contextualised is crucial for advancing evidence-based digital health coaching. This review followed PRISMA guidelines and was registered in PROSPERO (Registration number: CRD42022363279). The Irish Research Council supported this work.

**Systematic Review Registration:**

https://www.crd.york.ac.uk/PROSPERO/view/CRD42022363279, identifier: CRD42022363279.

## 1 Introduction

The United Nations (UN) has developed a universal agenda for sustainable development, with the overarching vision for all human beings to thrive and to reach their full potential in dignity and equality in a healthy environment (1). The third goal (SDG3) of this agenda is to “ensure healthy lives and promote wellbeing for all at all ages” (1). Under this goal, the UN emphasised reducing noncommunicable disease (NCD)-related mortality rates by one-third through prevention and treatment and promoting mental health and wellbeing (1). Digital health has been identified as a feasible, accessible and affordable solution to the UN goals of preventing NCDs and promoting health and wellbeing (2). Digital health is a complex and multifaceted field of both knowledge and practice, focused on the development and use of various technologies (i.e., smart watches, digital tracking tools, robotics, artificial intelligence and machine learning) for improving health (2–4). There has been substantial growth in the field of digital health research, especially in terms of evidence-based digital health interventions (DHIs) (5).

DHIs create new opportunities for medical doctors, health professionals and other caregivers to scale and tailor health and lifestyle interventions at a lower cost (5). DHIs are particularly effective for promoting healthy lifestyle behaviours like healthy eating, physical activity, stress reduction and psychological wellbeing (6–10). These behaviours, in turn, can promote healthier living and aid in the prevention and management of lifestyle-related NCDs such as cardiovascular disease, diabetes, certain cancers and chronic respiratory diseases (4, 5, 11–13). However, challenges exist in DHI implementation, particularly with self-guided or automated interventions. Despite their low cost and scalability, these interventions are often perceived as shallow, impersonal, and transactional, with participants preferring human support for more meaningful engagement (4, 14–17). Retention and engagement also pose significant issues, with a pooled estimated dropout rate of 43% across DHIs (18–20).

Integrating health coaching with DHIs may offer a solution to the limitation of passive or automated interventions by providing live and engaging support to participants. Health coaching is a non-clinical, evidence-based health promotion intervention that promotes sustainable health behaviours and improves lifestyle outcomes through personalised, solution-focused, person-centred support. By tailoring interventions to individual needs and fostering a collaborative relationship, health coaching empowers clients to make informed decisions about their health and facilitates behaviour change (21–23). Health coaching is already recognised as a valuable asset to healthcare systems. Specifically, for the health promotion and prevention of NCDs (24, 25). As such, health coach-facilitated DHIs could create meaningful yet scalable and accessible digital health solutions for health promotion and NCD prevention (26–28).

This integration paves the way for a new field of research relating to digital health coaching. There has already been a substantial surge of professional coaches transitioning to digital spaces, with 93.3% of coaches globally transitioning to online coaching (29). In the digital space, health coaching is mediated through technology, where coaches interact with coachees via digital platforms to guide behaviour change (30). This allows coaches and clients to interact regardless of geographical location and through different modalities (i.e., through text, video call or phone call) (31). While the conversation between coaches and clients remains central to digital health coaching, it can be supported by a variety of technologies, including digital tracking tools (i.e., smart watches, glucose monitors), self-guided educational modules, habit tracking and automated reminders. Additionally, recent advancements in artificial intelligence (AI) have spurred research into conversational agent coaching or chatbot coaches as flexible, accessible and cost-efficient alternatives to human-facilitated digital health coaching (32, 33). Chatbots are AI-driven programmes designed to interact with individuals using natural language that mimics human dialogue through algorithm-generated responses (34). However, the active engagement between the coach (AI or human) and coachee through personalised and real-time interaction is central to digital health coaching.

While there has been increasing interest in the integration of coaching within DHIs, a clear gap remains in understanding the impact of different coaching modalities (e.g., human, AI, and hybrid) on participant engagement and health outcomes. Although some studies have investigated coach-facilitated DHIs, comprehensive analyses and synthesis regarding the effectiveness of these modalities, particularly their role in enhancing lifestyle behaviours and preventing NCDs, are limited. This systematic review aims to synthesise existing research on coach-facilitated DHIs to understand how digital health coaching is being used in DHIs and the impact it has on lifestyle outcomes and engagement. Ultimately, this review seeks to provide evidence to inform the development of future digital health interventions and research.

## 2 Materials and methods

The methodology for this systematic review complies with the Preferred Reporting Items for Systematic Reviews and Meta-Analyses (PRISMA) guidelines for systematic reviews (35) (Supplementary File S1). This protocol has been registered on PROSPERO (Registration number: CRD42022363279). After registration, amendments were made to enhance the comprehensiveness of the review. The review timeline was updated to incorporate a more recent search date. Additionally, further details were added to the inclusion criteria to clarify modifications. Lastly, the search strategy was revised to reflect the updated search parameters.

### 2.1 Inclusion and exclusion criteria

The PICO framework guided the definition of eligibility for this review (Table 1). However, in this review, the traditional “Comparator” element in PICO was replaced with “Context”, as this review focuses on the integration of coaching within DHIs without the need for a direct comparator.

**Table 1:** PICOS framework for study inclusion and exclusion.

PICOS heading	Inclusion and exclusion criteria
Population	Studies involving adults aged 18 or older, including adults with or at risk for NCDs (i.e., cancer, diabetes, cardiovascular disease). Studies involving children or adolescents were excluded
Intervention	DHIs delivered online through a web-based platform or mobile application tailored towards one or more of the six pillars of lifestyle medicine. DHIs were excluded if the DHI was a medical intervention or used as a part of medical or mental health treatments
Comparator	Studies that explored coach-facilitated DHIs with an active coaching component, meaning that there is two-way interaction between coach and participants via text, video, or phone calls. Non-interactive coaching interventions were excluded. This coaching component could be health, lifestyle, or wellbeing coaching delivered by a human coach or conversational agent. Studies focusing on in-person, career/executive, or athletic coaching were excluded
Outcomes	Studies were included if they reported at least one of the pillars of lifestyle as its primary outcome. Studies were excluded if their primary outcomes were clinical or biomedical
Study design	Only peer-reviewed studies were included, with no publication date restrictions. Non-English studies were excluded due to language limitations

This review seeks to gather evidence of coach-facilitated DHIs implemented to increase the health and wellbeing of populations. While this largely falls into preventative interventions to help reduce the risk of NCDs, substantial studies are using coach-facilitated DHIs to help increase the health and wellbeing of cohorts living with NCDs (predominantly cancers and diabetes). It would be remiss to exclude these cohorts because of their disease status, despite the intervention aligning with inclusion criteria. As a result, we included adults living with NCDs once the primary outcomes were focused on lifestyle outcomes and not clinical or medical outcomes or markers (i.e., HbA1c, Cholesterol, BMI, weight reduction or waist circumference). This review concentrated on the adult population (18 years and older). As such, studies involving adolescents or children were excluded.

Peer-reviewed studies were considered if they explored digital coaching in DHIs and addressed at least one of the lifestyle medicine pillars: sleep, physical activity, psychological wellbeing and stress management, substance use, or healthy eating. Studies were excluded if they focused on medical interventions or used coaching as a part of medical or mental health treatments (i.e., coaching for patient education, trust building for treatments, pain management, medication adherence) that were not directly aimed at increasing lifestyle or wellbeing outcomes. Similarly, studies with clinical primary outcomes or biological markers as the primary outcome were excluded.

Studies that incorporated human, AI or hybrid (AI and human) coaching components alongside DHIs were included. For the inclusion criteria, digital health coaching was defined as a coach-coachee partnership facilitated online with the purpose of providing tailored support to enhance the DHI and promote behaviour change and health outcomes among participants. The coaching component of the DHI must align with this definition, meaning that coaching through *ad hoc* non-interactive messages or notifications was excluded. Studies that explored in-person or face-to-face interventions only, as well as career, executive or athletic coaching, were excluded. Finally, all peer-reviewed studies from any country were included, and no publication date limit was applied, given the limited literature on coaching, especially digital coaching. Studies that were not available in English were excluded.

### 2.2 Search strategy

The search strategy used keywords identified through an initial review of the literature. Keywords were grouped using Boolean operators and truncations. The PICO Framework also guided the formation of the final search strategy (Table 2).

**Table 2:** Final search strategy using the PICO framework.

Population	Intervention	Context	Outcome
Adult*	“digital health” OR “eHealth” OR “mHealth” OR “mobile health” OR “digital intervention” OR “mobile application” OR “smartphone application” OR “health app” OR “internet intervention” OR “online program” OR “web-based intervention”	“coaching” OR “health coaching” OR “digital coaching” OR “lifestyle coaching” OR “motivational coaching” OR “chatbot” OR “artificial intelligence” “AI chatbot” OR “virtual coaching” OR “AI Coach” OR “automated coaching” OR “health coach” OR “coaching psychology” OR “digital health coach” OR “conversational agent”	“engagement” OR “adherence” OR “participation” OR “user engagement” OR “user adherence” OR “retention” OR “satisfaction” OR “user satisfaction” OR “acceptability” OR “usability” OR “lifestyle” OR “healthy lifestyle” “lifestyle change” OR “wellbeing” OR “healthy living” OR “behaviour change” OR “habit*” OR “Healthy eating” OR “physical activity” OR “nutrition” OR “weight management” OR “stress management” OR “sleep quality” OR “active minutes”
And not	“Adolescent*” OR “child*” OR “clinical outcome” OR “treatment” OR “rehab*” OR “athlet*” OR “sports coach*” OR “performance coach*” OR “clinical treatment”		

* denotes a truncation operator, used to search for word variations or endings.

### 2.3 Informational sources

We conducted searches in electronic databases, including APA PsychINFO, Medline, Web of Science, and Scopus, on February 1, 2025. In addition to the electronic database searches, backward and forward citation searching of included studies was conducted to identify any additional relevant studies. Furthermore, forward searching of protocols deemed relevant during screening was carried out to ensure the inclusion of studies that may have been missed in the initial search.

### 2.4 Study screening

Covidence, an online specialised systematic review website, was used to screen studies. One reviewer (CL) screened the titles and abstracts of the identified studies based on the eligibility criteria. Three reviewers (CL, ROD, and JL) then independently reviewed the identified full-text studies. Reviewers met to discuss and resolve any conflicts or disagreements. If consensus could not be reached, a fourth reviewer (PJD) was designated to assess the relevant records. Three reviewers are qualified health coaches accredited by the European Mentoring and Coaching Council (EMCC). Two reviewers hold psychology degrees (CL, ROD), and the fourth reviewer holds a PhD in immunology and a degree in counselling and psychotherapy (PJD). The third reviewer (JL) has a biomedical engineering degree with specific expertise in digital health research.

### 2.5 Data extraction process

Two reviewers (CL & ROD) independently extracted the data using the Covidence Data Extraction template. To resolve discrepancies in the data extracted, reviewers came together to review data extraction and any existing conflicts. Conflicts that couldn't be resolved were referred to a third reviewer for resolution (PJD). However, this was not needed. The following characteristics were recorded: author, year, study aim, participant description and inclusion and exclusion status, total participant number, study design, lifestyle focus, measures taken, key outcomes, coaching delivery and intensity, coaching theory, role of coaching, length of intervention, and limitations.

### 2.6 Quality assessment

The Mixed Methods Appraisal Tool assessed the quality of all studies included in the systematic review. This was conducted independently by two reviewers (CL and ROD), who then jointly reviewed independent quality appraisal for any conflicts. Unresolved conflicts were referred to a third reviewer (PJD), though this step was not needed. The final MMAT results are included in the supplementary files (Supplementary File S2).

### 2.7 Study synthesis

The identified studies were synthesised using a narrative synthesis in relation to the study question: How is digital health coaching used in DHIs, and what is the impact of lifestyle outcomes and engagement? The synthesis followed the steps outlined by Popay (36), which included: becoming familiar with the studies, organising them into logical categories, comparing and synthesising the studies, exploring the relationships within and between the studies and synthesising the data under the relevant themes. Studies included in this review were first grouped by coaching modality (i.e., human, AI, or hybrid coaching) and then further categorised by targeted lifestyle outcomes (physical activity, psychological wellbeing, stress management, healthy eating, sleep, and substance use) and engagement outcomes. We evaluated the consistency of the findings across studies to assess the certainty of evidence. Missing or inconsistent data that was not a criterion for inclusion (i.e., engagement statistics, coaching theories, and length of intervention) was noted during synthesis and presented in the results. Studies were not excluded based on these missing data, but it was considered as a limitation during synthesis. A meta-analysis could not be performed due to significant variations in sample populations, outcome measures, and study designs across the included studies.

## 3 Results

**Figure 1:**
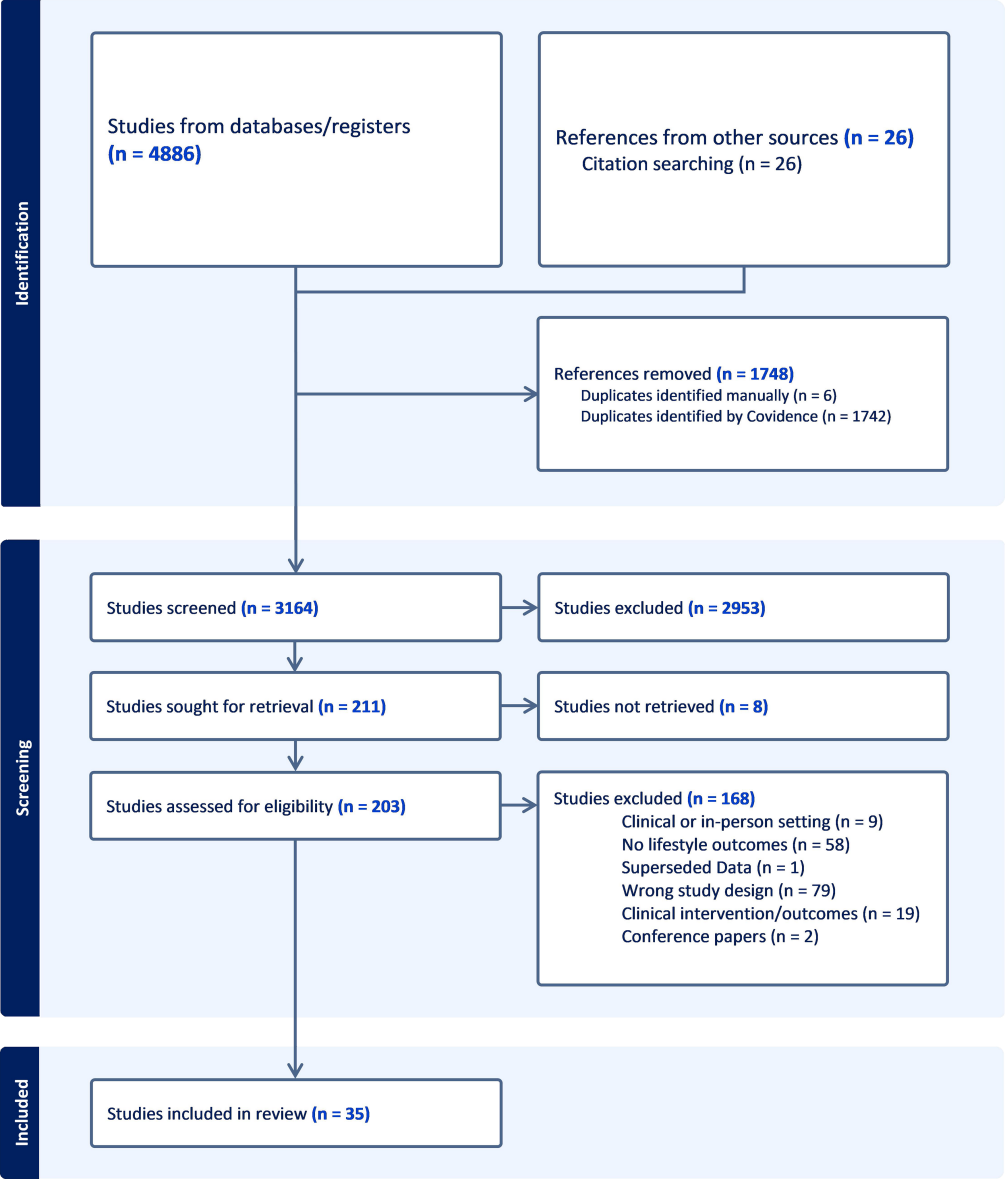
PRISMA flow diagram for systematic reviews illustrating the study selection process. Adapted with permission from “PRISMA 2020 flow diagram template for systematic reviews”, by Page et al., licensed under CC BY 4.0.

The database search yielded a total of 4,894 studies. After removing 1,748 duplicates, a further 2,936 studies were removed after title and abstract screening. One hundred and seventy-six studies were removed during the full-text review. In total, 35 studies were included in this review. The complete screening process is illustrated through the PRISMA diagram (Figure 1).

### 3.1 Quality assessment

No articles were excluded from the review based on the MMAT quality appraisal score (Supplementary File S2). Regarding the methodological rigour of the included articles, 11 studies scored 100% (5/5), and 23 scored 80% (4/5), indicating high quality. Only one study scored 60% (3/5), indicating medium quality. This demonstrates that the majority of studies included in the review met a high standard of methodological rigor.

### 3.2 Description of the included studies

Thirty-five studies were included for full review and data extraction. Table 3 summarises the study characteristics, including year of publication, country, length of intervention, number of participants, study design, intervention description, lifestyle areas addressed, measures used, and key outcomes. Most of the studies were pilot, feasibility, or early-stage studies, with only seven studies (20.6%) being full-scale randomised controlled trials (37–43). Intervention lengths ranged from 1 week to 12 months, and sample sizes ranged from 7 to 3,629 participants (Table 3).

**Table 3:** Characteristics of included studies.

Author	Year	Country	Length	Participants	Study design	Intervention	Lifestyle pillars	Measures	Outcomes
Alley et al. (44)	2,016	Australia	8 weeks	84 adults	Randomised controlled trial	Web-based physical activity intervention + video-based coaching	- Physical activity - Quality of life	Self-report measures: - Active Australia Survey (ASS) - SF-12 Health Survey	Increased physical activity: Tailoring + coaching (+150 min/week), Tailoring only (+123 min/week), Waitlist (+34 min/week). Significant mental health improvement in tailoring + video-coaching group only (*P* = .01)
Aymerich-Franch and Ferrer (17)	2022	Spain	3 weeks	32 adults	Nonrandomised controlled trial	Coaching programme delivered by a speech-based conversational agent (CAC)	- Psychological WB	Self-report measures: - Personal growth initiative scale (PGI) - Satisfaction with life scale (SLS) - Positive and negative affect scale (PANAS)	Significant increase in PGI (Pilot: *t* = 5.28, *P* = .013; main study: *t* = 3.84, *P* = .001). Moderate and significant increase in SLS (Pilot: *t =* 2.12, *P* = .124; main study: *t* = 4.99, *P* < .001). Moderate and significant decrease in negative affect (pilot: *t* = 2.37, *P* = .098; main study: *t* = 4.31, *P* < .001)
Bakas et al. (45)	2018	USA	3 weeks	22 older adults	Quasi-experimental design	A nurse-led intervention delivered through a telepresence robot for healthy independent living	- Psychological WB - Quality of life - Physical activity	- Self-report measures: - Unhealthy days measured as the sum of days in the past 30 days that physical and mental health had not been good. - Patient Health Questionnaire (PHQ-8). - Quality of life (0–10 scale) - Self-efficacy (6-item questionnaire) - Physical activity (number of minutes based on a 5-item questionnaire)	Intervention group saw a decline in unhealthy days (3.6 to 1.0 vs. control: 3.4 to 2.3). Slight decline in depressive symptoms (10.1 to 9.6 vs. control worsening to 10.6). Self-efficacy increased (8.2 to 9.1 vs. control: 8.8 to 8.4). Physical activity decreased slightly in both groups. Physical activity minutes declined for the intervention group (163.6 to 128.6 min/week) and slightly decreased for the waitlist control group (159 to 152.8 min/week)
Blair et al. (46)	2021	USA	13 weeks	54 older adults	Randomised controlled trial	Jawbone UP2 activity monitor paired with a smartphone app + tech support (control) or health coaching via phone call (intervention)	- Physical activity - Quality of life	Wearable device measures: - Total sedentary time - Number of breaks from sitting, - Standing time - Steps - Light and moderate intensity physical activity - Self-report measures: - Short Physical Performance Battery (SPPB) - Medical Health Outcomes Study Short Form 36-item survey (SF-36) - PROMIS Pain Interference Short Form - FACIT-Fatigue scale	No significant change in sedentary time: tech support group changed by 6.0 min (95% CI −39.5 to 51.6; *P* = .79), health coaching by 7.9 min (95% CI −30.8 to 46.6; *P* = .68). Steps/day increased significantly in the health coaching group (+1,675, *P* = .009) but not in the tech support group (+654, *P* = .37). Moderate-intensity physical activity increased significantly in the coaching group (*P* = .008), compared to control group (*P* = .33). No significant difference in SPPB scores
Chang et al. (47)	2023	USA	3 weeks	15 mothers	A single-group pre-test post-test study	Web-based lessons + health coaching sessions	- Healthy eating - Physical activity - Psychological WB - Stress management	Self-report measures: - NCI 5-factor screener - International Physical Activity Questionnaire-Short Form (IPAQ-SF) - Metabolic Equivalent Task (METs) units - Perceived Stress Scale (PSS-10) - Emotion Regulation Questionnaire - Treatment Self-Regulation Questionnaire - Healthy eating and self-efficacy (8-item survey) - Physical activity and self-efficacy (6-item survey - General Self-Efficacy Scale	Sugar intake decreased (21.07 to 12.53 tsp, *d* = 0.48, *P* = 0.126). Fruit/vegetable intake increased (4.73–5.55 cups, *d* = 0.49, *P* = 0.138). Physical activity showed minor improvement (107–171.5 METs, *d* = 0.13, *P* = 0.67). Stress reduced (18.33 to 14.67, *d* = -0.52, *P* = 0.097). Emotional control significantly improved (38.50–42.58, *d* = 0.71, *P* = 0.032). Motivation and self-efficacy improved across all domains. Motivation improved across all domains (healthy eating: *P* = 0.025; physical activity: *P* = 0.021; stress: *P* = 0.011). Self-efficacy increased for healthy eating only (*P* = 0.058)
Chew et al. (48)	2024	Singapore	1 week	251 adults	A single-group pre-test post-test study	AI–assisted weight management app with a chatbot-based check-in system, food-based computer vision image recognition and automated nudges	- Healthy eating - Physical activity - Psychological WB	Self-report measures: - Self-Report Habit Index (SRHI) - Consideration of Future Consequences Scale-6 items (CFCS-6) - Self-Regulation of Eating Behaviour Questionnaire (SREBQ) - IPAQ-SF - Generalised Anxiety Disorder Scale (GAD) - Patient Health Questionnaire-2 items (PHQ-2)	Statistically significant improvements in the overeating habit (*P* < .001), snacking habit (*P* = .02 - < .001), self-regulation of eating behaviour (*P* = .007), depression (*p* = .007), and physical activity (*P* < .001). Qualitative themes: increased self-monitoring, personalised reminders, food logging with image recognition, and engaging user interface
Chow et al. (27)	2020	USA	6 weeks	19 women cancer survivors	A single-group pre-test post-test study	iCanThrive, an app app-based intervention with eight educational modules + phone coaching	- Sleep - Psychological WB	Self-report measures: - The Centre for Epidemiologic Studies Depression Scale (CES-D), 10-item version - The Patient-Reported Outcomes Measurement Information System (PROMIS) - Self-Efficacy for Managing Emotions subscale (version 1.0, Short Form 4a)	Significant reduction in depressive symptoms (*t* = 2.22 and *P* = 0.04), slight increase in emotional self-efficacy (*t* = 1.33, *P* = 0.20), significant reduction in sleep disturbance (*t* = 3.41 and *P* = .003.). Continued significant difference in sleep disruption from baseline to the 4-week follow-up (*t* = 3.71; *P* = .002)
Daley et al. (49)	2020	Brazil	1 month	3,629 adults	A single-group pre-test post-test study	Vitalk, a mental health chatbot	- Stress management - Psychological WB	Self-report measures: - GAD-7 - PHQ-9 - Depression, Anxiety, and Stress Scale (DASS-21)	Significant reduction in depressive symptoms (*d* = 0.91, *P* = 0.001). 46.3% of users moved below the clinical cut off for PHQ-9. Significant anxiety reduction (*d* = -0.85, *P* = 0.001) and stress reduction (d = -0.81, *P* = 0.001), 49.0% of users moved from above to below the clinical cut-off for anxiety (GAD-7 score ≤ 8)
Damschroder et al. (37)	2020	USA	12 months	358 veterans	Randomised controlled trial	StayStrong app, activity monitoring using wearable devices + telephone health coaching	- Physical activity	Wearable device measures: - Active Minutes per week - Step count	Both groups showed a decline in active minutes (intervention: −41 min; control: −65 min) and step count (intervention: −1933; control: −2427). No significant difference between intervention and control at 12 months for active Minutes (*P* = .48) or step counts (*P* = .08)
D'Avolio et al. (50)	2023	USA	8 weeks	26 Dyads	Randomised controlled trial	Telehealth coaching program including nutritional education, stress-reduction material and coaching materials	- Healthy eating - Psychological WB	Self-report measures: - Mini-Nutritional Assessment (MNA) - 24-hour diet recalls collected on three days - Modified Caregiver Strain Index (MCSI) - PROMIS-Fatigue Questionnaire, - PROMIS Well-Being Questionnaire - SF-36	Protein intake increased significantly in the coached group (1.00 ± 0.17–1.35 ± 0.23 g/kg) vs. the not-coached (0.91 ± 0.19–1.01 ± 0.33 g/kg, *P* = .01, *η*² = .24). No significant effect of protein intake in FMWD. No significant changes in MCSI, SF36 physical component scale and mental component scale, or fatigue
Dhinagaran et al. (51)	2021	Singapore	4 weeks	52 adults	Single-arm feasibility study	Conversational agent promoting healthy lifestyle changes	- Physical activity - Healthy eating - Sleep - Quality of life	Self-report measures: - Adaptation of the food - Frequency questionnaire - IPAQ - Pittsburgh sleep quality index (PSQI) - PSS-10	Vegetable intake increased from 27% to 29% consuming vegetables at least once a day. Fruit intake increased from 3% to 7% (at least three portions). Participants who never consumed sweetened beverages increased from 38% to 45%. Participants who never consumed fried food and snacks increased from 25% to 30%. The stress score was reduced from 17 to 16. Sleep quality did not change significantly. Physical activity increased from 30 to 50 min/week. Time spent sitting reduced from 439 to 406, and METs per week score went from 857 to 765. Time in moderate to vigorous activity increased from 30 min per week at baseline to 50 min at follow-up
Foran et al. (38)	2024	USA	1 month	1,345 adults	Randomised controlled trial	Zenny—conversational agent designed to enhance wellbeing	- Psychological WB	Self-report measures: - World Health Organisation Wellbeing Scale (WHO-5 WB) - Flourishing scale - Mental health continuum-short form (MHC-SF)	Significant improvements in wellbeing (control: *d* = 0.24, *P* < .001; intervention: *d* = 0.26, *P* < .001), psychological flourishing (intervention: *d* = 0.19, *P* < .001; control: d = 0.18, *P* < .001), and positive psychological health (intervention: *d* = 0.17, *P* = .001; control: d = 0.24, *P* < .001). No significant differences in effectiveness between groups
Gabrielli (52)	2021	Italy	4 weeks	71 students	Proof of concept study	Atena—a psychoeducational chatbot supporting stress management	- Stress management - Psychological WB	Self-report measures: - GAD-7 - PSS-10 - Five-facet mindfulness questionnaire (FFMQ)	Significant reduction in anxiety symptoms (t39 = 0.94; *P* = .009), stress symptoms (t39 = 2.00; *P* = .05), and increase in mindfulness (*P* < .001)
Gudenkauf et al. (53)	2024	USA	8 weeks	13 caregivers	Single-arm pilot feasibility trial	DHI involving monitoring and visualising health-promoting behaviours and health coaching	- Physical activity - Sleep - Psychological WB	Self-report measures: - Health-Promoting Lifestyle Profile-II (HPLP-II) - European Organisation for Research and Treatment of Cancer Quality of Life Questionnaire (EORTC QLQ-C30) - PROMIS scales	Most participants showed improvement or maintenance of QOL (15% and 62%), sleep quality (23% and 62%), social engagement (23% and 69%), and general self-efficacy (23% and 62%). Physical activity outcomes were not reported
Han et al. (39)	2024	Singapore	6 months	148 adults	Randomised controlled trial	nBuddy Diabetes app, including features for diet, physical activity, behaviour change, blood glucose monitoring and health coaching	- Healthy eating	Self-report measures: - Changes in alternate healthy eating index-2010 (AHEI-2010) - 2-day food records	Significant improvement in diet quality by 6.2 points (95% CI, 3.8–8.7; *P* < .001) in the intervention group compared with control. Significant reduction in intake of sugar-sweetened beverages (−0.5 servings/day, 95% CI, −0.8, −0.2; *P* < .001) and sodium (−726 mg/day, 95% CI, −983, −468; *P* < .001)
Heber et al. (40)	2016	Germany	7 weeks	264 adults	Randomised controlled trial	Guided web and mobile-based stress management training + written feedback on every completed session from an e-coach	- Stress management - Psychological WB	Self-report measures: - PSS-10 - CES-D - Hospital Anxiety and Depression Scales (HADS-A) - Penn State Worry Questionnaire, Ultra Brief Version-past week (PSWQ-PW) - Short Form 12 (SF-12)	Large effects observed for perceived stress (*P* < .001, Cohen’s *d* = 0.83), depression (*d* = 0.95, *P* < .001), anxiety (*d* = 0.84, *P* < .001), and worry (*d* = 0.63, *P* < .001). Mental health scores improved significantly (*d* = 0.68, *P* < .001). Effects maintained at 12-month follow-up
Horn et al. (54)	2023	USA	12 months	26 women	Qualitative study	Balance after baby—web-based educational modules and lifestyle coaching	- Physical activity - Healthy eating	- Qualitative interviews	Participants reported changes in diet and physical activity as a result of the intervention. Online modules and support from the lifestyle coach were perceived to have had a positive effect on personal and familial lifestyle change. Other components were less utilised, including the community forum, gym memberships, and pedometers
Ly, Ly and Andersson (55)	2017	Sweden	2 weeks	28 adults	Randomised controlled trial	Smartphone app with an automated chatbot	- Stress management - Psychological WB	Self-report measures: - The Flourishing Scale (FS) - PSS-10 - The Satisfaction With Life Scale (SWLS)	Participants who adhered to the intervention (*n* = 13): significant effects for FS (F1, 27 = 5.12, *P* = 0.032), PSS-10 (F1, 27 = 4.30, *P* = 0.048). No significant effect for SWLS [F (1, 27) = 2.83, *P* = 0.10]. Within-group effects: PSS-10 [t (12) = 2.22, *P* = 0.046, d = 0.87] and SWLS [t (12) = -2.25, *P* = 0.044, d = 0.41] showed medium-to-large improvements in the intervention group
Maher et al. (56)	2020	Australia	12 weeks	31 adults	Case-control study	Artificial intelligence virtual health coach-led physical activity and diet intervention	- Physical activity - Healthy eating	Self-report measures: - 14-item Australian Mediterranean diet adherence tool - AAS	Participants increased physical activity by 109.8 min/week [F (2, 29) = 6.45, *P* = .005]. Mediterranean diet scores improved from 3.8 to 9.6 [F(2,29) = 44.56, *P* < .001]
Marler et al. (57)	2019	USA	18.5 weeks	319 adults	Prospective open-label single-arm study	Digital smoking cessation program incorporating a Food and Drug Administration, cleared carbon monoxide breath sensor, and text-based human coaching	- Smoking behaviours	Self-report measures: - Readiness to quit/stage of change - Confidence to quit, - Anticipated difficulty maintaining quit status - Smoking reduction - Quit attempts - 7-day and 30-day Point Prevalent Abstinence (PPA)	Positive changes in attitudes were observed from baseline to Mobilise (pre-Quit): increased confidence to quit (4.2–7.4, *P* < .001) and decreased expected difficulty maintaining quit (3.1–6.8, *P* < .001). Quit attempt rate: 79.4% (216/272, completer). 7-day PPA: 32.0%, 30-day PPA: 27.6%. 25.9% achieved ≥50% smoking reduction
McGuire et al. (58)	2022	Australia	8 Weeks	43 midlife adults	Two-arm parallel group feasibility study	GroWell for Health Program—eHealth intervention consisting of an interactive ebook and nurse coaching	- Healthy eating - Physical activity - Sleep - Substance use	Self-report measures: - IPAQ-SF - National Nutrition and Physical Activity Questionnaire - Readiness for change	Significant improvements in fruit intake, physical activity stage of change, and exercise habits for arm A (ebook and coaching) and arm b (ebook only) (*p* < .05).Alcohol frequency decreased for both groups, but this was not significant (arm A: 1.9 to 1.8 days a week, *p* = 0.65; arm B: 2.7 to 2.5 days a week, *P* = 0.62)
Moreno-Blance et al. (59)	2019	Spain	3 weeks	20 adults	Feasibility study	mHealth platform with an AI chatbot—Paola, educational material, recipes, diet and activity log	- Physical activity - Sleep - Healthy eating	Self-report measures: - Questionnaire monitoring adherence to a Mediterranean diet - Wearable device measures: - Physical activity - Sleep through	Participants met sleep goals 47.3% of days and logged moderate to vigorous activity 130% over the target. 10,000-step goal met 122% of the time. Adherence to a Mediterranean diet was not reported
Olliers et al. (60)	2023	Switzerland	Approx. 6 months	7,135 Adults	Single-arm interventional study	chatbot-led digital health app designed to provide lifestyle coaching across seven health areas to mitigate the collateral damage of the COVID-19 pandemic	- Psychological WB - Sleep - Healthy eating - Physical activity	- GAD-2 - PHQ - Insomnia severity index (ISI) - UCLA loneliness scale - Brief resilience coping scale - Single-item physical activity measure/international physical activity questionnaire short form - Short survey instruments for children's diet and physical activity	Significant decrease in anxiety levels between assessments [t(54) = 3.7, *P* < 0.001, d = 0.499; Intent-to-treat: t(416) = 3.4, *p* < 0.001, Cohen d = 0.165]. significant change in depression scores occurred (within group): F2,38 = 7.01, *P* = 0.003, with a large effect size. Physical activity, sleep and healthy eating did not report significant changes in outcomes between assessments (*P* = .847; *P* = .208; *P* = .837 respectively)
Partridge et al. (41)	2015	Australia	12 weeks	250 adults	Two-arm, parallel-group randomised controlled trial	Mobile app with education, self-monitoring and support resources, motivational text messages and coaching calls	- Healthy eating - Physical activity	Self-report measures: - IPAQ-SF - Short questions to assess SSB, fruit and vegetable intake and take-out meals	Intervention group increased vegetable intake (*P* = .009), reduced sugary drinks (*P* = .002), and improved physical activity (*P* = .05). They also increased their total physical activity by 252.5 MET-minutes (95% CI 1.2–503.8, *P* = .05) and total physical activity by 1.3 days (95% CI 0.5–2.2, *P* = .003) compared to controls
Price and Brunet (61)	2022	Canada	12 weeks	7 young adults	Single-arm feasibility trial	Telehealth behaviour change intervention	- Physical activity - Healthy eating	Self-report measures: - IPAQ-SF - Behavioural Risk Factor Surveillance System-FV questionnaire - Psychological Need Satisfaction in Exercise Scale - Psychological Need Satisfaction questionnaire - Exercise Treatment Self-Regulation Questionnaire - Dietary Self-Regulation questionnaire - Qualitative interviews	Participants reported significant increase in physical activity (T0 = 30; T1 = 150; *P* = 0.018), and fruit and vegetable intake (T0 = 2; T2 = 4.71; *P* = 0.018). There was also a significant increase in sense of autonomy (*P* = 0.046), competence (*P* = 0.018) and relatedness (*P* = 0.028) relating to fruit and vegetable intake, and a significant increase in autonomy (*P* = 0.027) and competence (*p* = 0.28) relating to physical activity. Qualitative data suggests the health coach created an autonomous supported environment, developed capacity for positive connections, and increased motivation
Sacher et al. (62)	2024	Netherlands	12 months	107 adults	Prospective single-arm study	Health coach-led, asynchronous, text-based, Digital Behaviour Change Coaching Intervention (DBCCI)	- Psychological WB - Healthy eating	Self-report measures: - Warwick-Edinburgh Mental Wellbeing Scale (WEMWBS) - GAD-7 - Loss of Control Over Eating Scale-Brief (LOCES) - Barriers to Being Active Quiz (BBAQ)	WEMWBS scores significantly improved at Month 6 and remained significant at Month 12 (*P* = .02). GAD-7 scores significantly decreased at Month 1 (*P* = .009). LOCES significantly decreased at month 1 (*P* < .001) and remained statistically significant at months 6 and 12 (*P* < .001). BBAQ scores significantly increased at Month 6 (*P* < .001) but did not remain significant at Month 12 (*P* = .45)
Santini et al. (63)	2023	Italy/Netherlands	10 weeks	91 adults	Longitudinal Mixed Methods Study	Digital coaching intervention	- Physical activity - Psychological WB	Self-report measures: - SF-12v2 Health Survey - IPAQ - WHO-5 WB - GSE-6 - Lubben 6-item Social Network Scale (LSNS-6)	Perceived worsened physical health (SF-12-PCS ≤50) increased from T0 to T2 (*p* = 0.002), while perceived mental well-being (SF-12-MCS ≤42) remained stable (*P* < 0.001). Physical activity significantly increased from T0 to T1, then stabilised (*P* = 0.012), with low activity rates decreasing from 8.2% to 6% during the human coach-supported phase but rising to 8.3% when using the system independently. Self-efficacy and socialisation levels fluctuated over time
Smart et al. (64)	2022	USA	8 weeks	28 adults	A single-group pre-test post-test study	Text-based coaching and Fitbit program	- Physical activity	Wearable device measures: - Steps - Physical activity minutes	There was no significant change in the average weekly steps (mean difference 7.26, SD 6209.3; *P* = .99), sedentary minutes (mean difference −17.6, 95% CI −67.8 to 32.6), or light (mean difference −3.37, 95% CI −28.8 to 22.1) and moderate to vigorous physical activity (mean difference 6.79, 95% CI −3.4 to 17.0)
Spring et al. (42)	2018	USA	9 months	212 adults	Randomised controlled trial	Multicomponent intervention integrating mHealth, modest incentives, and remote coaching	- Healthy eating - Physical activity	Self-report measures: - Moderate to vigorous active minutes - Dietary data was recorded through the CalorieKing food database	Sustained improvement in composite diet and activity score at 3, 6, and 9 months (*P* < .001). Sequential treatment showed slightly greater improvement at 6 months (*P* = .03), but no difference at 3 or 9 months compared to simultaneous treatment
Terblanche et al. (65)	2022	South Africa	6 months	168 students	Randomised controlled trial	AI Chatbot Coach	- Stress management - Psychological WB	Self-report measures: - Goal attainment scale - WEMWBS - Brief Resilience Scale (BRS) - PSS-10	Psychological wellbeing, resilience and stress showed a medium-sized correlation with each other, ranging from *r* = .50 to *r* = -.64 (correlations with stress being negative). The experimental group showed an increase of 55% on their goal attainment compared to 24% in the control group. No statistical significance was found between psychological wellbeing, resilience, stress and time and group (resilience, *P* = 0.80; psychological wellbeing, *P* = 0.89; and stress, *P* = 0.91)
To, Green and Vandelanotte (66)	2021	Australia	6 weeks	116 adults	Quasi-experimental design without control group	Physical activity chatbot and a connected wearable device	- Physical activity	Self-report measures: - AAS - Wearable device feedback: - Step count	Participants reported an increase in steps from baseline (increase of 627, 95% CI 219–1035 steps/day; *P* < 0.01) and total physical activity (increase of 154.2 min/week; 3.58 times higher at follow-up; 95% CI 2.28–5.63; *P* < 0.001). Participants were also more likely to meet the physical activity guidelines (odds ratio 6.37, 95% CI 3.31–12.27; *P* < 0.001) at follow-up
Ulrich et al. (67)	2024	Switzerland	7 weeks	230 students	Randomised controlled trial	Conversational agent–delivered stress management coaching intervention	- Stress management - Psychological WB	Self-report measures: - PSS-10 - PHQ-9 - PHQ-15 - GAD-7 - Active coping (5-point Likert scale) - General Self-Efficacy Scale (GSES)	Significant reduction in perceived stress (Cohen *d* = -0.60, *P* = .001), depressive and psychosomatic symptoms (Cohen d = -0.50, *P* = .003; Cohen d = −0.36, *P* = .010). No significant change in anxiety and active coping (Cohen d = −0.29, *P* = .08; Cohen d = 0.13, *P* = .06)
Wiegand et al. (68)	2010	USA	14 weeks	562 women	Longitudinal Study	Internet-based online coaching, education programme and olfactive-based personal care products	- Stress management - Psychological WB	Self-report measures: - PSS-10 - Profile of Mood States (POMS) - Trier Inventory of Chronic Stress (TICS) - Spielberger State-Trait Anxiety Inventory (STAI) - St Mary's Hospital Sleep Questionnaire (SMS) - Short-Form-36 (SF-36) - Work Productivity and Activity Impairment Questionnaire (WPAI)	Group 1 (coaching, education programme, and olfactive-based products) had statistically significant improvement in the PSS score vs. Group 3 (control) (*P* < 0.01). Group 2 (coaching and education programme) demonstrated significantly greater reductions vs. baseline (*P* < 0.001), but there were no statistically significant differences vs. Group 3. Significant improvements in other efficacy outcomes such as POMS total mood disturbance, TICS work overload and social responsibility subscales, STAI and awakenings, (*P* < 0.05)
Wijsman et al. (43)	2013	Netherlands	3 months	226 older adults	Randomised controlled trial	Philips DirectLife: monitoring and feedback by accelerometer and digital coaching	- Physical activity	Wearable device measures: - Active minutes	At the ankle, activity counts increased by 46% [standard error (SE) 7%] in the intervention group, compared to 12% (SE 3%) in the control group (*P*=<.001). Measured at the wrist, activity counts increased by 11% (SE 3%) in the intervention group and 5% (SE 2%) in the control group (*P* = .11). After processing of the data, this corresponded to a daily increase of 11 min in moderate-to-vigorous activity in the intervention group vs. 0 min in the control group (*P* = .001)
Williams et al. (69)	2021	New Zealand	21 days	64 students	Open trial single-arm study	Digital mental health intervention delivered by a chatbot	- Stress management - Psychological WB	Self-report measures: - WHO-5 wellbeing scale - Personal Well-being Measure (ONS4) - PSS-10 - GAD-7	WHO-5 scores improved significantly (SD = 15.07; *P* < 0.001). Mean reduction of the PSS-10 = 1.77 (SD = 4.69; *P* = 0.004) equating to effect sizes of 0.49 and 0.38, respectively. Those who were clinically anxious at baseline (*n* = 25) experienced a greater reduction of GAD-7 symptoms than those (*n* = 39) who started the study without clinical anxiety (1.56, SD = 3.31 vs. 0.67, SD = 3.30; *P* = 0.011)

To assess the heterogeneity of the participants, demographic information, including sex, age, race, and education, was collected and is presented in Table 4. Participants were predominantly Caucasian, though African American/Black, Asian, multiracial, and other racial groups were represented in smaller proportions. Notably, four studies had a larger representation of African American/Black (47) and Asian participants (39, 48, 51). The mean age of participants varied widely. Participants were largely aged between 40 and 60 years, with the exception of outlying studies focusing on younger adults and students (18–34 years) (47–49, 51, 52, 55, 61, 65, 67, 69) and older populations (60 + years) (38, 43, 45, 46, 50). Women were more frequently recruited for digital health interventions, with female participation ranging from 25.2% to 100% across studies. Education levels were inconsistently reported, but in studies that provided this information, the percentage of participants with a college degree ranged from 20.7% to 95%.

**Table 4:** Participant demographics.

Author	Gender %female	Age (mean)	Age (range)	Race %					College degree (%)
				Caucasian	African American/Black	Asian	Multiracial	Other	
Alley et al. (2016) (44)	76	54.21							41.66
Aymerich-Franch and Ferrer (2022) (17)	53.3	40.47							76.7
Bakas et al. (2018) (45)	71.4	84		100					
Blair et al. (2021) (46)	56	69.6		57				7	57
Chang et al. (2023) (47)	100	32.9		33.33	46.66		6.66	13.33	60
Chew et al. (2024) (48)	52	31.25				97		3	95
Chow et al. (2020) (27)	100	59.6		94	3		3		
Daley et al. (2020) (49)	76		18–24						
Damschroder et al. (2020) (37)	25.2	39.8		64.7	13.2			22.1	93.6
D'Avolio et al. (2023) (50)	92.3	66.2		84.6	3.8		3.8	7.7	
Dhinagaran et al. (2021) (51)	62	33.7		3		93		2	87
Foran et al. (2024) (38)	80.3	47.17							
Gabrielli et al. (2021) (52)	67.6		18–34						
Gudenkauf et al. (2024) (53)	61.5	52	28–70	69.2	23.1			7.7	76.9
Han et al. (2024) (39)	40	53.1				92.5		7.5	
Heber et al. (2016) (40)	73.1	43.3		83.3				16.7	76.9
Horn et al. (2023) (54)	100	34.5		65	23	12	23		62
Ly, Ly and Andersson (2017) (55)	53.5	23.3							
Maher et al. (2020) (56)	67.7	56.2							
Marler et al. (2019) (57)	57.7		30–39	82.8	6.9	1.6			81.5
McGuire et al. (2022) (58)	89.2	50.6							77.7
Moreno-Blanco et al. (2019) (59)	33.30	30							
Ollier et al. (2023) (60)	71.3	46.3							
Partridge et al. (2015) (41)	27.7	61.3							80.6
Price and Brunet (2022) (61)	85.7	33.8							71.4
Sacher et al. (2024) (62)	89.7	41.8		59.9	1.9	10.3			
Santini et al. (2023) (63)	35.5								46.8
Smart et al. (2022) (64)	77	47.1		3	80			17	60
Spring et al. (2018) (42)	76.4	40.8		41	46.7	3.8		8.5	69.3
Terblanche et al. (2022) (65)	56	22							
To, Green and Vandelanotte (66)	81.9	49.1		87.1				12.9	
Ulrich et al. (2024) (67)	73.6	26.7							26.4
Wiegand et al. (2010) (68)	100	35.7		68.3	21.7			9.7	20.7
Wijsman et al. (2013) (43)	40.9	64.8							56.7
Williams et al. (2021) (69)	81		18–23						2.4

### 3.3 Presence of coaching in DHIs

This review focused on the role and impact of health coaching in DHIs. Table 5 summarises the key characteristics of coaching that are evidenced across included studies. Coaching was exhibited through two crucial elements: (1) an active coaching component and (2) evidence-based coaching theories. Theories not only guided the coaching process (27, 40–42, 45, 46, 50, 53, 57, 58, 61, 62, 68) but also influenced the overall design of DHIs and AI chatbots (17, 38, 48, 49, 51, 52, 55, 56, 60, 65–67, 69). The active coaching component of DHIs was evident through three different delivery modes: (1) digital human coaching, (2) AI-powered coaching, or (3) a mix of both, which we refer to as hybrid coaching (Table 5). Using the three modes of coaching identified, this review presents the features of coaching, followed by the trends of lifestyle outcomes and engagement that emerged from the included studies.

**Table 5:** Characteristics of coaching.

Author	Coaching approach	Theories/frameworks	Coach qualification disclosed	Number of sessions	Frequency of sessions	Session length	Mode of communication**^b^**	Role in digital health
Alley et al. (2016) (44)	Hybrid	- Behaviour change theory (BCT) - Communication theory	No	4	Bi-weekly	10–15 min	Synchronous	Supportive
Aymerich-Franch and Ferrer (2022) (17)	AI	- SMART coaching model - Quality of Life Therapy	N/A^a^	3	Weekly	10–14 min	Synchronous	Central
Bakas et al. (2018) (45)	Human	- Lorig's evidence-based Living a healthy life - Chronic Conditions Toolkit10	Yes	3	Not stated	Not stated	Synchronous	Central
Blair et al. (2021) (46)	Human	- Social Cognitive Theory (SCT) - Behaviour Change Theory	No	5	Not stated	15–20 min	Synchronous	Supportive
Chang et al. (2023) (47)	Human	- Not stated	No	3	Not Stated	Not stated	Synchronous	Central
Chew et al. (2024) (48)	AI	- Self-regulation theory - Behaviour change taxonomy	N/A^a^	Not stated	Not stated	Not stated	Asynchronous	Central
Chow et al. (2020) (27)	Human	- Efficiency Model of Support - Cognitive Behavioural Therapy (CBT) - Acceptance-based therapy - Positive psychology	No	2	Monthly	30 and 5 min	Synchronous/asynchronous	Supportive
Daley et al. (2020) (49)	AI	- CBT - Positive Psychology	N/A^a^	4–5	Not stated	5 min	Synchronous	Central
Damschroder et al. (2020) (37)	Hybrid	- Information-motivation-behavioural skills (IMB) model - Self-regulatory theory	No	3	Over 9 weeks	30 min	Synchronous	Supportive
D'Avolio et al. (2023) (50)	Human	- Goal-attainment theory - Personal change and development	No	8	Weekly	45–60 min	Synchronous	Central
Dhinagaran et al. (2021) (51)	AI	- Capability, Opportunity, Motivation, Behaviour model (COM-B)	N/A^a^	Not stated	Not stated	Not stated	Asynchronous	Central
Foran et al. (2024) (38)	ÀI	- BCT - Goal-attainment theory - Positive Psychology	N/A	Not stated	Daily	Not stated	Asynchronous	Central
Gabrielli et al. (2021) (52)	AI	- CBT - Positive psychology	N/A^a^	8	Bi-weekly	10 min	Synchronous	Central
Gudenkauf et al. (2024) (53)	Human	- Health-Promoting Lifestyle Profile (HPLP-II) checklist	Yes	8	Weekly	15–20 min	Synchronous	Central
Han et al. (2024) (39)	Human	Not stated	Yes	Not stated	*ad hoc*	Not stated	Asynchronous	Supportive
Heber et al. (2016) (40)	Human	- Lazarus's transactional model of stress	Yes	8	After educational sessions	Not stated	Asynchronous	Supportive
Horn et al. (2023) (54)	Human	- Not stated	Yes	24	Weekly for 12 weeks, bi-weekly for 12 weeks and monthly thereafter	Not stated	Synchronous	Supportive
Ly et al. (2017) (55)	AI	- CBT - Positive psychology	N/A^a^	Not stated	Not stated	Not stated	Synchronous	Central
Maher et al. (2020) (56)	AI	- BCT	N/A^a^	Unlimited	Unlimited	Not stated	Note stated	Central
Marler et al. (2019) (57)	Human	- 5 A's (Ask, Advise, Assess, Assist, Arrange) - Cognitive behavioural therapy - Motivational interviewing - Self-determination theory	No	Not stated	3 times weekly for the first 30 days, 1 weekly for the next 30 days, and biweekly for the last 30 days	Not stated	Asynchronous	Central
McGuire et al. (2022) (58)	Human	- Transtheoretical model of health behaviour change - Motivational interviewing	Yes	3	Week 1, 4, and 8	30–60 min	Synchronous	Supportive
Moreno-Blanco et al. (2019) (59)	Hybrid	- Not stated	No	Not stated	Not stated	Not stated	Synchronous asynchronous	Central
Ollier et al. (2023) (60)	AI	- Health Action Process Approach - Positive psychology coaching - Motivational interviewing - CBT	N/A	Not stated	Not stated	5–10 min	Synchronous	Central
Partridge et al. (2015) (41)	Human	- Transtheoretical model of change	Yes	5	Not stated	10–25 min	Synchronous	Central
Price and Brunet (2022) (61)	Human	- Self-determination theory - Motivational Interviewing - BCT	No	12	Weekly	60 min	Synchronous	Central
Sacher et al. (2024) (62)	Human	- BCT - COM-B - Theoretical domains framework	Yes	Not states	Not stated	Not stated	Asynchronous	Central
Santini et al. (2023) (63)	Hybrid	Not stated	No	Not stated	Daily	Not stated	Asynchronous/synchronous	Central
Smart et al. (2022) (64)	Human	- SMART goals	Yes	Not stated	Not stated	Not stated	Asynchronous	Central
Spring et al. (2018) (42)	Human	- BCT	Yes	22	Weekly (week 1–12), bi-weekly (13–24), monthly (25–40)	10–15 min	Synchronous	Central
Terblanche et al., (2022) (65)	AI	- Goal-attainment theory - GROW model - DAIC framework	N/A^a^	Unlimited	Unlimited	Not stated	Synchronous	Central
To et al. (66)	AI	- Health Action Process Approach - CBT - Mindfulness	N/A^a^	12	Every 2 to 4 days	Not stated	Synchronous	Central
Ulrich et al. (2024) (67)	AI	- COM-B	N/A^a^	Unlimited	Unlimited	Not stated	Asynchronous	Central
Wiegand et al. (2010) (68)	Human	- BCT	No	Not stated	Not stated	Not stated	Synchronous	Central
Wijsman et al. (2013) (43)	Human	- Not stated	No	Not stated	Not stated	Not stated	Asynchronous	Central
Williams et al. (2021) (69)	AI	- CBT - Positive Psychology	N/A^a^	21	Daily	3–5 min	Synchronous	Central

^a^ Not Applicable for AI studies.

^b^ Mode of communication is catagorised into synchronous, meaning real-time communication between coach and participant, and asynchronous, meaning communication which is not time-bound or instant.

#### 3.3.1 Digital human coaching

Digital human coaching refers to any study that used a human coach alongside DHIs. Eighteen of the included studies (51%) employed human coaching as a part of their interventions (27, 39–43, 45–47, 50, 53, 54, 57, 58, 61, 62, 64, 68). Coaching was often enhanced with digital tracking tools (e.g., smart scales, watches, accelerometers, and breath sensors) (41–43, 46, 53, 57, 58, 62, 64) and educational resources or modules (27, 39–41, 47, 50, 54, 58, 61, 62, 68). One study used a telepresence robot controlled remotely by practitioners to facilitate communication with participants (45). A consistent finding emerged from these studies: the human coach played a crucial role in engaging participants and enhancing motivation, adherence, behaviour change, and personalisation (39, 46, 58, 61, 62). However, the intensity of human coaching delivered varied. In most cases (67%), the coach was central to the intervention, interacting regularly with participants. In contrast, six studies assigned coaches a more supportive role, primarily supplementing educational modules, training, and activity monitors (27, 39, 40, 46, 54, 58). In these instances, participants primarily engaged with the digital components and the coaches provided additional support to reinforce engagement and adherence, focusing only on issues related to the main intervention (27, 39, 40, 46, 54, 58).

#### 3.3.2 AI-powered coaching

Thirteen included studies (37%) analysed the use of AI-powered coaching using conversational agents (i.e., chatbots) to deliver DHIs (17, 38, 48, 49, 51, 52, 55, 56, 60, 65–67, 69). The development of conversational agents and chatbots in these studies was primarily based on Natural Language Processing (NLP) models (17, 49, 51, 56) or rule-based approaches, including decision-tree algorithms (38, 51). Most AI coaching interventions were delivered remotely through a digital platform, available 24/7 (38, 45, 48, 49, 51, 52, 55, 56, 60, 65, 66, 69). However, one study used on-site AI coaching in a controlled environment (17). Eleven studies mimicked human coaching sessions through quick text-based sessions with the AI chatbot. One chatbot, however, was primarily voice-based, with text-based options for certain activities. This chatbot could interpret speech and synthesise voices to respond to participants (17). Three studies provided unlimited access to the AI chatbots, enabling *ad hoc* questions, as well as guidance, monitoring and feedback outside of structured coaching sessions (56, 65, 66). Unlike human coaches, AI coaches were central to all DHIs. Given the artificial nature of the coaching design, no coaching qualifications or accreditations were reported in AI coaching studies.

#### 3.3.3 Hybrid coaching

Four studies (11%) investigated hybrid coaching in DHIs (37, 44, 59, 63). Hybrid coaching refers to any study that integrates human coaches with AI-powered features within a DHI. Three studies investigated the impact of combining human coaching (delivered via video or text) with automated personalisation, support, advice, and motivation delivered by in-app messages, nudges or knowledge pills (short tips or pieces of advice) (37, 44, 59). The AI in these studies typically performed administrative tasks such as (a) sending personalised interventions and educational content (44, 59, 63), (b) monitoring activities, progress, engagement and adherence (37, 63), (c) sending motivational messages or nudges (37, 63), and (d) administering questionnaires (59). While two studies used AI and human coaching in parallel, the third study employed a “human-in-the-loop” approach for delivering hybrid coaching, where the human coach monitored, modified, and validated the AI coach's recommendations (59). The fourth study combined human coaching with a digital coach (similar to AI-powered coaching). The digital coach was an avatar that participants could interact with in-app through text-based prompts (63). This resulted in the AI-powered coach taking on more interactive and meaningful tasks like goal-setting with participants (63).

### 3.4 Lifestyle outcomes across coaching modalities

The reviewed studies assessed changes in physical activity, psychological wellbeing, stress management, healthy eating, sleep, and substance use across human-delivered, AI, and hybrid coaching interventions (Table 3). Among these lifestyle domains, physical activity was the most commonly addressed, covered by 55.8% of studies (*n* = 19), followed by psychological wellbeing (*n* = 18; 52.9%). Stress management was examined in nine studies and was closely associated with psychological wellbeing, with many studies addressing both constructs together. Given its interrelated role, stress management was discussed as a subset of psychological wellbeing in this review. Healthy eating was explored in 13 studies, while sleep (*n* = 4) and substance use (*n* = 2) were less commonly examined.

#### 3.4.1 Physical activity

Physical activity was assessed through a variety measures, including active minutes, Metabolic Equivalent Tasks (METs), daily steps, or sedentary time measured through biometric feedback (37, 42–44, 59, 64, 66) and self-reported physical activity assessments (41, 45–48, 51, 56, 58, 61, 63, 66), and qualitative interviews (54). Studies examining human coaching reported mixed findings regarding its impact on physical activity. Several studies found improvements in physical activity measured through changes in METs (41, 47) and moderate-to-vigorous activity (MVPA) minutes (42, 43). However, other studies showed no meaningful changes or a decline in physical activity (45, 46, 64). Some studies also found that human-coach-facilitated DHIs promoted shifts in participants' awareness, motivation, and readiness to change, leading to active change in physical activity (46, 47), along with an increase in purposeful movement, like taking the stairs or walking instead of driving (54).

AI coaching generally showed consistent positive effects on physical activity. Studies reported improvements in physical activity measures, including METs (48), step count (66), active minutes (56, 66), MVPA minutes, and reduced sitting time (51). AI coaching was also associated with improved adherence and increased motivation (56). All four hybrid studies investigated the impact of this approach on physical activity, reporting variable results. Two studies found significant to moderate improvements in physical activity through active minutes (44, 63). However, a third study found decreased active minutes and steps among both intervention and control groups (37). The fourth study did not assess changes in physical activity from baseline but did report high rates of adherence and goal completion (i.e., achieving step targets and weekly active minute goals) (59).

#### 3.4.2 Psychological wellbeing

Psychological wellbeing was primarily assessed through the reduction of depression and anxiety symptoms (27, 40, 45, 49, 52, 60, 62, 67) and general psychological wellbeing using a variety of wellbeing and mental health scales (50, 53, 62, 63, 65, 69). Other aspects of psychological wellbeing, such as worry, emotional control, mindfulness, personal growth, and life satisfaction, were also assessed to a lesser extent (17, 27, 47, 52, 55). Human coaching interventions were associated with reductions in depressive and anxiety symptoms (27, 40, 45, 62), along with reduced worry (40) and increased emotional self-efficacy and emotional control (47, 62). However, this was not reflected in one study among coached and non-coached groups (50) and another which aimed to improve health and wellbeing but did not use scales to assess wellbeing changes (53). AI coaching generally reported positive improvements in psychological wellbeing, including reductions in depressive symptoms (49, 60, 67) and anxiety (49, 52, 60), as well as improvement in general psychological wellbeing (69). AI coaching interventions also improved other wellbeing constructs like mindfulness (52), personal growth (17, 55), and life satisfaction (55). However, two AI-coaching interventions failed to report differences in psychological wellbeing (65, 67). Only one hybrid coaching study assessed psychological wellbeing (63]. While psychological wellbeing improved from baselines, this was not sustained after the human-coaching element of the intervention ended (63).

##### 3.4.2.1 Stress management

All human coaching interventions examining perceived stress reported positive outcomes (40, 47, 68), with some showing greater improvement compared to control groups (40, 68). However, one study observed only minimal reductions in stress levels from baseline (47). Likewise, AI coaching interventions generally led to reductions in perceived stress, ranging from significant (52, 55, 67, 69) to minor improvements (49). One AI intervention reported no positive changes in stress levels (65). Hybrid coaching interventions did not assess stress management outcomes.

#### 3.4.3 Healthy eating

Healthy eating was examined in AI and human coaching interventions only. The most common measure used to assess dietary improvements was daily fruit and vegetable intake (41, 51, 58, 61). Other measures included overall diet quality assessed via questionnaires (39, 42), adherence to the Mediterranean diet (56), protein intake (50), behaviours related to overeating and self-regulation (48). Intervention utilising human coaching demonstrated significant improvements in protein intake (50), fruit and vegetable consumption (41, 58, 61), and overall diet quality (39, 42). Two studies explored behavioural factors influencing healthy eating, reporting increased autonomy and competence (61) and improved control over eating habits (62). AI coaching interventions also yielded positive effects on healthy eating, though to varying degrees. While all three studies showed positive changes (48, 51, 56), only one found significant improvement in their diet scores (56). The latter study focused on eating habits rather than food quality, reporting significant reduction in overeating and snacking habits as well as self-regulation in eating behaviour (48).

#### 3.4.4 Sleep

Sleep was assessed through self-reported sleep quality (27, 51, 53), and sleep duration measured via wearable devices (59). Studies examining human coaching generally reported improvements in sleep quality. One study found that most participants either improved or maintained their sleep quality (53), while another reduced sleep disturbances, with improvements sustained at follow-up (27). In contrast, AI coaching showed minimal impact, with no significant improvements in sleep quality or sleep scores (51). Hybrid coaching interventions provided limited findings on sleep. While one hybrid study assessed sleep outcomes, baseline comparisons were not available. However, results indicated that participants met the recommended seven to eight hours of sleep on nearly half of the recorded days (59).

#### 3.4.5 Substance use

Substance use was assessed by two studies using human coaching. Both studies showed that coaching interventions had a positive impact on decreasing alcohol consumption (58), tobacco use, and smoking quit rates (57). The human-coach-led smoking cessation intervention also enhanced participants' confidence to quit smoking and reduced perceived difficulty in maintaining abstinence (57).

### 3.5 Engagement and satisfaction

Engagement was measured inconsistently across studies, with definitions and measures varying widely (Table 6). The most commonly reported metric was retention or intervention completion rate. For human coaching interventions, completion and retention rates varied from 80% to 100% (27, 39–41, 46, 47, 53, 57). AI coaching interventions reported similar completion and retention rates of 90% to 93%, with three outlying studies reporting significantly lower completion rates of 58% (52), 20.3%–45.4% (49), and 9.8% (60). Hybrid coaching interventions had retention rates of 55%–56.5% (37, 44). However, one hybrid study found that the human coaching component improved adherence to syncing data from wearable devices (37).

**Table 6:** Engagement and satisfaction.

Author	Engagement	Satisfaction
Alley et al. (2016) (44)	Total Completion rate: 55% (83/151). 47% completed ≥3 modules, coaching completers: 82% vs. 43% others. Retention: No group difference. Week 9 survey completion: 73% (coaching) vs. 53% (others). Average website visits: 7.53; Average time spent: 87.07 min. Participants who completed coaching spent significantly more time on the website (174.64 min vs. 77.84 min)	68% satisfied with the program; 77% with website usability; 76% with tailored advice; 91% with module questions. Coaching completers reported higher satisfaction (88% vs. 64%, not significant)
Aymerich-Franch and Ferrer (2022) (17)	Completion rate: 92% (30/32 participants)	Medium-high satisfaction with the coaching program. satisfaction with the coaching program at 6.06 out of 7
Bakas et al. (2018) (45)	N/A	N/A
Blair et al. (2021) (46)	79% participants checked the app daily. 93% completed all 5 coaching calls. Retention rate: 87% (47/54 participants)	N/A
Chang et al. (2023) (47)	Retention rate—80% (12 of 15 participants). All participants attended all three online health coaching sessions	N/A
Chew et al. (2024) (48)	Completion rate: 91.6%	N/A
Chow et al. (2020) (27)	The app was launched 21.5 times over 6 weeks. Completion rate: 87%	Mean satisfaction: 5.19/7. Usefulness of coaching calls: 4.22/5
Daley et al. (2020) (49)	Completion rate: 20.34%–45.4%, depending on the program. Higher engagement correlated with lower anxiety and depression. Average response rate: 8.17 responses/day	N/A
Damschroder et al. (2020) (37)	Engagement was high initially but declined. 64.4% provided synched data at 6 months, 35.6% at 12 months. 70.8% completed ≥2 coaching calls, 56.7% completed all 3 calls. The coaching group correlated with better adherence to synched data (68.5% vs. 60.3%, difference not sustained)	N/A
D'Avolio et al. (2023) (50)	N/A	N/A
Dhinagaran (2021) (51)	Completion rate: 93%. 50% of participants completed all conversations. 40% responded immediately 75% of the time	92% moderately satisfied. 54% likely to recommend, 57% likely to use again
Foran et al. (2024) (38)	Participants engaged with the intervention 4.26 days over 30 days, started 3.68 modules, completed 2.78, and sent 51.09 messages on average	3.21/5 satisfaction score with modules. User satisfaction and participation/engagement were significantly associated with greater improvements in all primary outcomes (*P* = .04 to < .001). Participants with more unfinished modules (modules started but not completed) showed less improvement in positive psychological health
Gabrielli et al. (2021) (52)	Completion rate: 86% (61 out of 71 participants). By the end of the study, 58% (41 out of 71) of participants completed the postintervention questionnaire, representing an attrition rate of 42%. Engagement and willingness to complete a session were higher during the first and last weeks of the study	N/A
Gudenkauf et al. (2024) (53)	Participants completed 6.9/8 weekly assessments and attended 6.9/8 coaching sessions (86.5%). Wore wearable device 79.9% of study days. 100% baseline completers did an 8-week follow-up	Satisfaction: 4.7/5. 85% rated satisfaction as 5/5
Han et al. (2024) (39)	Completion rate: 95%. App utilisation: 87% (first 3 months), 92% (4–6 months). Two-way interaction: 3 days/week (first 3 months), 2 days/week (4–6 months)	N/A
Heber et al. (2016) (40)	Participants completed 5.7/7 sessions (81.4%) and used the intervention for 8.27 weeks. 43.6% of participants preferred light coaching interaction, and 56.4% preferred intensive coaching. 76.5% requested message support	92.2% satisfied with overall intervention
Horn et al. (2023) (54)	N/A	N/A
Ly et al. (2017) (55)	During the 2-week period, 78.6% of participants were active for at least 50% of the days. Active for more than half of the interventions 14 days. (average 8.21 days). Participants opened the app 1.27 times a day	N/A
Maher et al. (2020) (56)	Out of the maximum of 11 possible check-ins with chatbot, participants completed an average of 6.9 check-ins (64%). Engagement varied across the intervention. 70% of participants completed check-ins in weeks 2, 3, 4, and 12. Engagement gradually decreased to around 50% through weeks 8 and 9. Participants who completed the first weekly check-in had higher engagement across the intervention period than those who didn’t. completion rate was 90% (28/31)	N/A
Marler et al. (2019) (57)	Retention rate: 97.3% (183/188 participants). Completion rate: 95.2% (179/188 participants). The intervention group opened the app an average of 157.9 (vs. 86.5 in control, *p* < .001) times. High weekly login rates: 86%-98% (intervention), 85%–97% (control)	N/A
McGuire et al. (2022) (58)	The nurse coaching group had better adherence and lower attrition (35% vs. 50%) compared to the eBook group	N/A
Moreno-Blanco et al. (2019) (59)	Users read 88.09% of knowledge pills and reported following advice for 65.9%	Usability score (SUS): 81.5. indicating usability and high satisfaction
Ollier et al. (2023) (60)	Completion of at least one topic: 9.8% (*n* = 698). 7,135 downloaded the app, and 3,928 opened the app (55.8%)	The net promoter score increased as individuals progressed between periods 1 and 2. Ease of use and usefulness also increased. However, only marginally
Partridge et al. (2015) (41)	Completion rate: 85.6%. The mean number of coaching calls completed was 4.6/5, with 82.4% completing all 5 calls. Over half of the intervention participants replied to 8 or more of the 16 SMS text messages, with 20.3% replying to all. Most control participants replied to 2 or more of the 4 text messages, with 62.4% replying to all 4	N/A
Price et al. (2022) (61)	The engagement for this intervention was high, with participants attending 95.2% of the sessions. Session attendance ranged between 66.7% (8 out of 12 sessions) and 100% (12 out of 12 sessions)	N/A
Sacher et al. (2024) (62)	Out of the 122 eligible participants who provided consent, 119 were enrolled, and 107 were included in the analysis	81.9% found the health coaching useful/helpful
Santini et al. (2023) (63)	Completion rate: 68% (62/91 participants)	Average SUS score: 59 (below the average score of 68), indicating usability problems with the system
Smart et al. (2022) (64)	73% set at least 7 goals over 8 weeks, and 47% set goals every week. Coaches sent 3–4 more messages/week than participants. Completion rate: 93% (28/30)	N/A
Spring et al. (2018) (42)	Retention at 9 months: 82.1%. Self-monitoring adherence declined but remained substantial (96.3% at baseline, 54.6% at 9 months). Coaching calls declined from 66.0% to 57.7%	N/A
Terblanche et al. 2022 (65)	Experimental group retention rate: 56% (75 out of 134), and for the control group: 70% (94 out of 134). Participants who used the AI coaching chatbot more frequently (more than 6 sessions) had a higher average increase in goal attainment (37.62) compared to those who used it less frequently (17.62)	N/A
To et al. (66)	60% of the intervention group completed the post-intervention survey. 45% completed all 13 sessions. Engagement ratio: 74.3% (297 responses/400 messages)	N/A
Ulrich et al. (2024) (67)	On average, participants sent 6.7 messages per week to the chatbot and spent. Most participants (93.8%) read the messages sent by the chatbot. About half of the participants sent messages to the chatbot at least once a day	The average usability score for the chatbot was 61.6, with the majority of participants rating the chatbot as “OK” (78.8%) or “Good” (10.6%). Less than half of the participants (43.4%) would recommend the chatbot to others
Wiegand et al. (2010) (68)	N/A	N/A
Wijsman et al. (2013) (43)	Completion rate: 97% (226/235 participants). 95.6% of participants in the intervention group started the program after the initial assessment week. 91.2% of participants in the intervention group completed the 12-week intervention program	N/A
Williams et al. (2021) (69)	Completion rate: 27.3% (30/110 participants). Adherence: 11/21 days (M = 11.3, SD = 7.8)	Satisfaction: 6.61/10 (SD = 1.78), 63% rated ≥7/10. 81% found chatbot easy to use

Several studies reported correlations between engagement levels and outcomes. Studies investigating the addition of a human-coaching component in DHIs found that participants who completed coaching spent more time on the DHI, completed more educational modules (44), had better adherence to the intervention (37, 58), improved retention (61) and increased wellbeing (63). Additionally, higher engagement with AI chatbots correlated with lower anxiety and depressive symptoms (49), improved wellbeing (38), increased physical activity (66), and a higher increase in goal attainment (65). Finally, one study found that early engagement (within the first week) predicted sustained engagement throughout the intervention (56), while another reported that participants who completed the full intervention had lower stress levels (40).

#### 3.5.1 Satisfaction

Satisfaction with the DHI interventions was reported in 37% of the included studies (*n* = 13) (Table 6). Among hybrid studies, 75% (3 out of 4) reported satisfaction outcomes. However, AI coaching interventions had the highest proportion of studies reporting satisfaction, accounting for 46% (6/13), compared to 22% (4/18) of human coaching studies. Overall satisfaction rates were high across studies using human coaching in their DHIs, with 81.9% to 92.5% of participants reporting satisfaction for coaching interventions (40, 62). Additional studies reporting satisfaction through mean scores reported that 85% of participants rated a full score for satisfaction (5/5) (53) and a mean reporting score of 5.19/7 (27). AI coaching interventions showed mixed satisfaction outcomes, generally ranging from moderate to high satisfaction rates (17, 38, 51, 69). Three studies reported on the likelihood of participants recommending the intervention. One study found a significant change in the Net Promoter Score (60), while two studies reported that 43.4% and 54% of participants would recommend the AI intervention. Additionally, 57% of participants indicated they would use it again (51, 66). Hybrid studies reported varied satisfaction rates varying from 67% to 81.5% (44, 59), with one study reporting usability below the average threshold, indicating low satisfaction (63). Interestingly, one hybrid study found that satisfaction rates were higher among participants who completed the coaching component of the intervention (88% compared to 64%) (44).

### 3.6 Working alliance

Seven studies (20.6%) examined working alliance and connection between participants and coaches. While one study used a working alliance scale, most explored working alliance through qualitative feedback from participants. Human coaching showed a high working alliance. Participants experienced authentic and strong connections, social support, and accountability (64). They also described a sense of investment and warmth from their coach (61), even in text-based coaching (64). This allowed participants to feel comfortable, motivated, and honest about their progress (61). Participants perceived AI coaches as engaging and lifelike, often viewing their interactions as relational (55, 60, 69). Participants viewed chatbots as a positive addition, appreciating its non-judgemental nature, finding it easier to share information (17) and feeling validated in their experiences (69). On a 7-point working alliance scale, the overall alliance with AI and human coaches was rated 4.23, with the bond component scoring 4.20 (67). Another study using the Session Alliance Inventory (ISA) found a minor but insignificant increase in scores (60). Despite positive connections made, some participants found chatbots patronising and preferred to connect to a real person. Others reported feelings of loneliness, disconnection and a lack of warmth while engaging with chatbots (17, 69). One study noted that chatbot interactions felt repetitive, contributing to feelings of disconnection (55). In one hybrid coaching intervention, participants valued human support alongside the AI coaching and expressed a desire for continued human support alongside the AI interventions, particularly for motivation, confidence, and technical support (63).

## 4 Discussion

This review explored how digital health coaching is integrated into DHIs and its impact on lifestyle outcomes and engagement. We identified three primary coaching models: human, AI, and hybrid (a combination of both human and AI coaches). Our findings suggest that both human- and AI-delivered coaching are generally perceived as acceptable and satisfactory components of DHIs, with trends indicating positive effects on health and wellbeing. Engagement and retention were generally high across all coaching models, with higher engagement linked to improvements in lifestyle outcomes. However, engagement and satisfaction were typically higher with human-delivered coaching. While working alliance was strong across all coaching models, participants reported a stronger sense of connection with human-delivered coaching, including within hybrid interventions.

Despite advancements in digital health coaching, studies were predominantly exploratory, early-intervention studies, focusing on feasibility and acceptability of coach-facilitated DHIs. While individual studies reported significant findings, the lack of consistency in study designs and outcome measures prevented clear inferences about broader trends in lifestyle and engagement. Likewise, there was an imbalanced representation of human (*n* = 18), AI (*n* = 13), and hybrid (*n* = 4) coaching interventions. This disparity, along with inconsistencies in outcome measures, intervention designs and coaching characteristics, limited direct comparisons and the generalisability of our findings; therefore, caution is warranted when interpreting the results in this review.

### 4.1 The role of coaching: delivery, standards, and trends

This review examined the integration of health coaching with DHIs, focusing on delivery methods, coaching roles, and standards for protocols and coach engagement (Table 5). Findings revealed a lack of consistency in reporting delivery methods, which contributed to ambiguity in the coaching protocols used. Only six studies provided comprehensive descriptions of all the extracted coaching characteristics that represent delivery (17, 42, 52, 53, 58, 69). The lack of transparency in coaching delivery methods made it difficult to determine the optimal frequency and intensity of coach-participant engagement for best outcomes. The total number of coaching sessions varied widely, ranging from 2 to 24, with a mean of 8.85 sessions per intervention (Table 5). Additionally, coaching sessions were most commonly conducted weekly (17, 50, 53, 61) and biweekly (44, 52), but no clear pattern emerged linking coaching frequency to delivery mode. Among long-term studies (ranging from 3 to 12 months), coaching was typically staggered, starting with weekly coaching sessions before transitioning to bi-weekly and monthly as the intervention progressed (42, 54, 57). Very few studies reported the qualifications of the coaches, making it difficult to determine who was delivering the coaching. Nonetheless, some trends emerged from the studies that did provide details on coaching delivery methods. Most studies indicated that both AI and human coaches played a central role in DHIs, though eight studies employed coaches in a supportive capacity (27, 37, 39, 40, 44, 46, 54, 58). In these cases, the role of the coach typically focused on supporting participants' technology use and enhancing usability through education around app features (27, 37, 46), reviewing and monitoring participant data (46), providing feedback to participants (39, 40, 44), adding accountability and promoting the implementation of skills gathered from the main intervention (i.e., educational modules and training) (27, 44, 54, 58).

In several studies, the role of the coach extended beyond the traditional role of facilitating health behaviour change and providing lifestyle support (27, 37, 46). Coaches were often tasked with providing technological *and* lifestyle support. The burden of supporting participants in navigating DHI technology has been recognised (27, 37). However, only one study provided a separate technology support channel for participants (46). In this instance, technology support was carried out by coaches via telephone calls but conducted outside of the coaching sessions. Moreover, communication predominantly occurred synchronously, with human coaches or conversational agents speaking in real time with participants. However, some studies supplemented this mode of interaction with asynchronous communication between coaching sessions (i.e., check-ins and *ad hoc* questions and feedback) (27, 59, 63).

#### 4.1.2 Coaching standards

Professional and accredited coaches should play a central role in the development of digital health coaching interventions, whether by directly providing coaching or by informing the creation of coaching chatbots. This ensures that the high standards of practice paved by professional bodies like the International Coaching Federation (ICF), the European Mentoring and Coaching Council (EMCC) and the Association for Coaching (AC) are upheld in DHI research. In this review, we identified two key components for maintaining standards: (1) the qualifications and training of the coaches involved and (2) the evidence-based theories and models that inform coaching practices in DHIs. Only ten studies reported the qualifications or training of the human coaches involved in the interventions (39–42, 45, 53, 54, 58, 62, 64). These coaches were typically dietitians (39, 41), nutritional coaches (54), psychologists (40, 64), nurse practitioners (45, 58), and trained health coaches (53). Other studies mentioned that coaches received training from qualified professionals to deliver the intervention (42, 62). None of the hybrid coaching studies reported the qualifications or accreditation of the coaches working alongside AI.

Likewise, coaching theories played a crucial role in underpinning DHIs across all coaching models. Cognitive behavioural therapy, behaviour change theories, and positive psychology were the most commonly used (Table 5). All AI coaching interventions presented theories that underpinned the development of their conversational agents. Although six studies using human coaches failed to report theories used, two of which were hybrid coaching interventions (43, 47, 53, 54, 59, 63). This suggests that coaching theories were not only vital for active coaching but also influenced the design of DHIs and AI chatbots. The use of validated, evidence-based theories in the development of chatbots demonstrates that studies are moving towards validated, evidence-based approaches for chatbot creation. However, it also underscores the need for standardised quality and ethical guidelines for AI coaching.

The Designing AI Coach (DAIC) framework developed by Terblanche (70) provides a structured approach for assessing AI standards in coaching. The framework emphasises adapting human-efficacy elements and theoretical models for specific and narrow coaching tasks (i.e., goal setting), in line with ethical codes of conduct from accrediting coaching bodies (i.e., ICF, EMCC, AC). In this review, we found that all AI coaching studies, either intentionally or unintentionally, followed the first two principles of the DAIC framework: adapting human-efficacy and incorporating theoretical models in chatbot development (Table 5). However, the third principle, ethical conduct (covering privacy, autonomy, liability, and bias), was only addressed in three studies (49, 55, 65). The fourth principle, using AI chatbots for narrow, specialised tasks, was met in all but two studies. Notably, Maher et al. (56) used AI for both nutritional and physical activity interventions. Likewise, Dhinagaran et al. (51) and Ollier et al. (60) extended their chatbot's role to cover a wide range of lifestyle areas. Dhinagaran et al. (51) included diet, exercise, sleep, and stress support, while Ollier et al. (60) focused on psychological well-being, healthy eating, physical activity, and sleep. The DAIC framework is a valuable framework for guiding the development of AI coaching chatbots. Further research and standardisation are essential to ensure that AI coaching aligns with ethical and professional standards. This is critical for ensuring high-quality and safe AI interventions in digital health.

### 4.2 Tends in engagement, satisfaction, and working alliance

Engagement and retention rates have traditionally been considered low for DHIs (71), suggesting that this is a common issue in digital health research. However, a recent review by Boucher and Raiker (72) argued that engagement rates are not inherently low but rather vary widely across studies. They also highlighted a high degree of variability in how engagement is defined and measured (72). Our findings align with those of Boucher and Raiker (72), revealing that engagement was inconsistently reported, causing limitations for comparisons across studies. Retention and completion rates were the most consistently reported metrics. These metrics inferred higher retention and completion rates for human coaching interventions compared to AI and hybrid interventions. While AI retention rates were similar to those for human coaching, hybrid interventions showed a comparable difference. Engagement metrics offered valuable insight into adherence and retention but did not clarify the nature of meaningful interactions that may influence adherence. Boucher & Raiker (72) recommended moving away from the one-size-fits-all approach to assessing engagement and instead encourage researchers to focus on patterns of engagement.

Engagement as behaviour (i.e., quantitative engagement measures like app logins, text messages sent, and coaching sessions attended) provides valuable insight into the dynamics of DHIs and engagement trends (73). However, this construct was also inconsistently reported across studies, making it challenging to draw definitive conclusions (Table 6). Engagement as behaviour suggested that engagement with both human and AI coaches yielded positive outcomes. Among studies that reported engagement as behaviour, trends indicated early engagement and increased engagement with both human and AI coaches led to better outcomes, increased retention, adherence and goal attainment (37, 38, 40, 44, 49, 56, 58, 61, 63, 65, 66). Satisfaction rates offered further insight into participants' experiences with different coaching modalities. Overall, satisfaction was highest in interventions using human-facilitated coaching interventions (27, 40, 53, 62). AI coaching showed more variable satisfaction rates (17, 38, 51, 66, 69). While hybrid approaches demonstrated higher rates of satisfaction over AI-only, they still lagged behind human-only interventions (44, 59, 63).

Participants' experiences were further uncovered through the exploration of working alliances developed between coaches and participants. Prior research has consistently linked strong working alliances with positive coaching outcomes (74). A positive working alliance was formed between participants and AI and human coaches during DHI studies (17, 55, 60, 61, 63, 64, 67, 69). However, AI chatbots reported limitations to the alliances formed, with some participants reporting a sense of disconnection, repetitiveness and lack of warmth with chatbots (17, 55, 69). This trend in working alliance, favouring human-facilitated coaching interventions, was reflected by one hybrid coaching intervention (63). This study reported that participants valued human support along with AI to increase motivation and confidence and support technology navigation (63). Overall, while engagement, satisfaction, and working alliance remained consistent across both human and AI coaching interventions, human-facilitated coaching interventions foster stronger connections and higher satisfaction. Although hybrid and AI models could promote engagement and offer positive participant experiences, they could not replicate the interpersonal elements of human-delivered interventions that enhanced the overall individual experience. While engagement remains a key indicator of adherence, Boucher and Raiker (72) suggested that the true measure of success in DHIs should focus on the ability to foster meaningful lifestyle changes rather than being based on the frequency or duration of engagement.

### 4.3 Effectiveness of coaching for lifestyle change

The review findings suggest that human and AI-facilitated coaching could produce positive effects on lifestyle to varying degrees. While AI-powered coaching demonstrated more consistent positive trends on physical activity levels (48, 51, 56, 66), human-facilitated coaching yielded more consistent positive outcomes for psychological wellbeing and stress management (27, 40, 45, 47, 62, 68). Likewise, both human and AI coaching reported overall improvements in healthy eating (39, 41, 42, 48, 50, 51, 56, 58, 61, 62). Human coaching reported improvements in sleep, whereas AI did not (27, 51, 53, 59). Finally, substance use was only assessed with human coaches, precluding direct comparisons (57, 58). However, the varying effects of lifestyle outcomes between human and AI delivery systems were minimal, indicating that both AI and human coaching are acceptable and feasible interventions for supporting lifestyle changes. This finding aligns with existing literature suggesting that AI can be equally effective in providing support, particularly when deployed for narrow, specific tasks (32). Such findings suggest that AI could be a promising avenue for delivering scalable and effective lifestyle interventions (75, 76). However, studies using hybrid coaching approaches failed to produce consistent, robust findings compared to AI- and human-only interventions. Although some hybrid studies showed promising effects in physical activity, psychological wellbeing, and sleep, one study indicated that these benefits did not persist after human support ended (63). This suggests that further refinement of the integration between human and AI elements is needed to optimise long-term behaviour change.

### 4.4 Exploring the hybrid AI-human coaching model: benefits, limitations, and future potential

The lack of significant engagement and lifestyle outcomes in hybrid studies was unexpected. AI pioneers have established the value and complementary nature of combining AI and humans (77), with a growing body of literature on AI-human symbiosis indicating the shifting division of work between humans and machines (77–79). This comes from longstanding research asserting that computers plus humans do better than either alone (77, 79, 80). AI and humans hold different values and capabilities. While AI has superior computational and analytical skills, humans can better deal with uncertainty and have greater aptitude for intuition, creativity, and holistic work (77, 79). By harnessing human-AI symbioses, it becomes possible to pull the best traits of AI and humans and compensate for the limitations of both (77, 80).

In digital health coaching, hybrid models have been proposed as a promising way to combine the scalability of AI with the relational depth of human coaching (31, 81). This hybrid approach draws on the strengths of AI and human coaches to create high-quality, meaningful, accessible, and scalable coaching conversations (31, 81). Despite this theoretical promise, hybrid coaching studies included in this review did not reflect this. Several factors may explain this, including methodical constraints, the limited role of human coaches, and inconsistencies in AI implementation. Firstly, only four studies in this review examined hybrid coaching, making it difficult to assess its full impact. Secondly, human coaching was commonly offered as a supportive or optional component of hybrid DHIs (37, 82). Three studies highlighted this as a limitation, acknowledging greater outcomes when coaching was the central component of DHIs (37). Participants also expressed a preference for periodic human interaction alongside AI-driven support, reinforcing the importance of sustained human involvement (44, 63). Furthermore, these studies indicated that consistent human support was important for maintaining improvements in lifestyle and sustaining engagement (37, 44, 63). This aligns with existing literature, which suggests that AI should extend rather than replace human coaching capabilities (83).

The AI components used in hybrid coaching varied across studies. Two studies employed basic automation for delivering tailored messages and advice (37, 44), while another used a decision-support system to provide personalised recommendations (59). The fourth study integrated a digital coach similar to a chatbot but with limited interactive capabilities (63). The study that most closely aligned with AI coaching models showed significant improvements in physical activity; however, these benefits diminished once human support was removed (Table 3). This reflects broader findings that AI is effective for structured tasks like goal-setting but lacks the relational depth and working alliance needed for long-term behavioural change (83). This suggests that hybrid coaching is still an emerging area with limited empirical evaluation. While these studies confirmed the feasibility, more research is needed to refine hybrid coaching models and optimise their effectiveness. Additionally, trends suggest that hybrid models should prioritise human connection as the foundation of coaching while leveraging AI to enhance efficiency and scalability. Future hybrid interventions should, therefore, prioritise AI as an enhancement to human coaching rather than a substitute.

### 4.5 Strengths and limitations

This systematic review provided a comprehensive exploration of coach-facilitated digital DHIs, a novel and growing field. By synthesising existing evidence, this review highlighted the feasibility, acceptability, and impact of human, AI and hybrid coaching modalities on engagement and lifestyle outcomes. Adherence to PRISMA guidelines and the use of the MMAT ensured a rigorous and transparent methodological approach. There are well-established, foundational reviews that have significantly advanced digital health research in the context of health promotion and disease prevention (6, 84, 85). This review builds on this existing foundation to synthesise the existing body of knowledge on the integration of different health coaching modalities within DHIs. The insights gained from this review can inform the development and optimisation of future coach-led DHIs.

However, several limitations of this review should be noted. First, while full-text screening, data-extraction and quality appraisal was done in duplicate, the title and abstract screening was conducted by one reviewer only, increasing the risk of reviewer bias. Additionally, due to the use of broad search terms with multiple definitions, it is difficult to confirm that all relevant articles have been included. Furthermore, Significant heterogeneity in reporting coaching protocol, engagement metrics, and lifestyle outcomes across studies affects the ability to compare findings directly. This heterogeneity hindered the synthesis of results, making it difficult to draw consistent conclusions. Many of the included studies primarily focused on assessing the acceptability and feasibility of coach-facilitated DHIs rather than investigating the impact of specific components of the DHIs (e.g., active coaching, educational modules, tracking tools) and their interactions in influencing lifestyle outcomes. As a result, this review cannot definitively establish the full impact of coaching within DHIs, and its findings should be interpreted with caution. The review's generalisability is also constrained by the heterogeneity of participant demographics. Finally, the inclusion of only peer-reviewed studies may have introduced publication bias, potentially skewing the findings. Despite these limitations, this review offers valuable insights into the current state and potential future directions of digital health coaching in DHIs, providing a foundation for advancing scalable, high-quality, and evidence-based interventions.

## 5 Conclusion

The studies included in this review contribute to our understanding of how digital health coaching can be effectively integrated into DHIs, highlighting the trends, opportunities, and challenges related to its impact on lifestyle outcomes and engagement. Despite inconsistencies in reporting coaching delivery methods, engagement metrics, satisfaction, and lifestyle outcomes, this review emphasises the potential positive impact of coach-facilitated DHIs on participant engagement and lifestyle outcomes. We confirm the acceptability and feasibility of integrating AI and human coaching into DHIs and identify a gap in the research on hybrid coaching approaches. Digital health coaching in DHIs is complex and multifaceted, making it difficult to isolate components of the intervention to understand their effect (i.e., coaching, wearables, environment). Further research is necessary to better understand these complexities to advance the development of quality, evidence-based, coach-led DHIs that promote participant engagement and positive lifestyle changes. Additionally, it is crucial for future research to explore how the benefits of both AI and human coaching, as reported in this review, can be leveraged through hybrid approaches. Such strategies could help overcome the barriers of meaningful engagement in AI coaching and the scalability and accessibility of human coaching to create a scalable coach-led DHI without decreasing the quality of health coaching.

## Data Availability

The original contributions presented in the study are included in the article/Supplementary Material, further inquiries can be directed to the corresponding author.
